# PERVading strategies and infectious risk for clinical xenotransplantation

**DOI:** 10.1111/xen.12402

**Published:** 2018-08-18

**Authors:** Christopher G. A. McGregor, Yasu Takeuchi, Linda Scobie, Guerard Byrne

**Affiliations:** ^1^ Institute of Cardiovascular Science University College London London UK; ^2^ Department of Surgery University of Alabama Birmingham Birmingham AL USA; ^3^ Department of Cardiovascular Surgery Mayo Clinic Rochester MN USA; ^4^ Division of Infection and Immunity University College London London UK; ^5^ Division of Advanced Therapies National Institute for Biological Standards and Control South Mims UK; ^6^ School of Health and Life Sciences Glasgow Caledonian University Glasgow UK

**Keywords:** infectious risk, PERV

The recent improvements in efficacy and survival of pre‐clinical renal,^1^, ^2^ islet,^3^, ^4^ and cardiac^5^ xenotransplantation have reinvigorated interest in clinical xenotransplantation. This renewed interest makes it essential for clinicians, regulators, and the general public and potential patients to have a clear understanding of the risk represented by porcine endogenous retrovirus (PERV). PERV is a unique infectious risk for xenotransplantation because it is carried as part of the porcine genome. Unlike exogenous viruses, microorganisms, and parasites, PERV cannot be excluded by cesarean birth or the high health, intensive husbandry methods which do exclude these other pathogens from designated pathogen‐free (DPF) barrier‐derived pigs. The potential risk of PERV infection for humans was first identified in 1997 when porcine PK15 cells^6^ and later NIH minipig cells^7^ were shown to infect human HEK293 cells in culture. Shortly after this discovery, calls were made by some^8^ but not others^9^ to place a moratorium on ongoing clinical xenotransplantation trials. This led to a revision of FDA guidelines for xenotransplantation which effectively banned the use of non‐human primate tissues, reflecting the more serious infectious concerns that non‐human primate material presents. The renewed guidelines also required establishing procedures and assays to monitor the potential for PERV infection when implanting porcine tissue. Since that time, extensive investigation into the basic virology of PERV has occurred and numerous assays developed,^10^ much of which are discussed in this issue of *xenotransplantation*. What is clear with respect to PERV is that all pigs are not created equal and the circumstances of putative PERV infectivity must be considered in any discussion.

The critical concern for clinical xenotransplantation is whether the donor organ will be infectious to the recipient human patient, their family or caregivers, or the general population. If transplanted cell tissues or organs contained cells with the retroviral properties of PK15 or were derived from most, but not all, minipigs,^11^, ^12^, ^13^, ^14^ the frequency of PERV infection in vitro for primary human cells is demonstrable,^7^, ^15^, ^16^ suggesting at least the potential for clinical infection. Post‐operative infection, however, may not occur even with these tissue sources as in vitro testing excludes the significant impact of innate and adaptive immunity at least some of which, such as preformed antibody and complement, will be active even in immune‐suppressed patients. If however the donor tissue is from a known analyzed agricultural pig strain, such as the Large White, Landrace, or Duroc pigs,^17^, ^18^, ^19^, ^20^ then PERV infection of human cells, even under the most permissive in vitro conditions, has not resulted in productive infection. A high genetic deficiency of PERV provirus loci, estimated to range from 10 to 100 copies, exists between individual pigs and pig strains.^16^ Indeed, the porcine reference genome, derived from a Duroc pig, encodes 20 PERV sites without large deletions, but all of them are defective and incapable of producing a functional virus.^21^ The number of clinical xenotransplantation studies is necessarily limited, but both retrospective and prospective studies of patients exposed to pig tissues have failed to find evidence of PERV infection.^22^, ^23^, ^24^, ^25^, ^26^, ^27^, ^28^, ^29^, ^30^ It is important to recognize that some PERV literature which describes both pig‐to‐human and human‐to‐human PERV infection is in reference to in vitro studies, using known infectious cell lines, and does not represent clinical infection of patients. Thus, from a clinical perspective, there has never been a documented case of pig‐to‐human or human‐to‐human PERV infection.

Pigs which are not able to infect HEK293 cells or primary human cell in vitro appear to share certain characteristics, a reduced frequency of human‐tropic PERV‐A and PERV‐B sites, PERV sites with lower levels of RNA synthesis and a high frequency of sequence degeneracy. Pigs lacking the porcine‐tropic PERV‐C virus are also advantageous as they are incapable of producing PERV‐A/C recombinants which exhibit a higher human tropism and replication rate in human cells. Animals with these characteristics can be readily identified within the agricultural strain background and using current PCR screening and next‐generation sequencing methods thoroughly characterized and monitored. Recently, the CRISPR/Cas9 gene‐targeting method has been applied to PERV to engineer deletion/insertion mutations within the viral polymerase gene.^31^ This new technology further reduces the potential of PERV infection and recombination, but the frequency of karyotype anomalies raises new concerns of unforeseen genomic changes.^32^ The live birth of CRISP/Cas9 PERV polymerase‐engineered pigs, derived from PERV‐C‐negative fibroblast with no known PERV infectivity, is encouraging, but further analysis of these animals is necessary to exclude such unanticipated genetic effects.^32^

A degenerate constellation of PERV sites, naturally occurring or engineered, does not mean that the chance of infection from these tissues is zero, as recombination between different PERV sites, between PERV and other porcine endogenous retroviruses,^33^ or between PERV and human retroviruses could theoretically result in a functional virus, but if it occurred would be at low frequency^34^ with minimal risk in clinical xenotransplantation. Selecting porcine donor tissue with fully degenerate PERV sequences does however reduce the in vitro frequency of infection from these tissues and thus is expected to proportionately reduce the likelihood of in vivo infection. If such an event occurred, in vitro studies have shown that human‐tropic PERV is susceptible to antiviral therapies,^35^, ^36^, ^37^ adding a prophylactic layer of therapeutic control to the donor preventative considerations described above.

UNOS estimates that 20 people die each day on the transplant waiting list. This human loss is however an underestimate of the need for transplant organs as the chronic shortage of donor organs means that many patients who would benefit from transplantation are never placed on to the waiting list. In the last 20 years, a wealth of information on PERV and other porcine zoonotic pathogens has been generated resulting in the development of DPF barrier facilities, assays to monitor infectious zoonotic pathogens, including PERV, preventative strategies to severely limit the likelihood of PERV infection, and identification of therapeutics to treat the potential infection. While no single method can fully eliminate the theoretical risk that PERV presents, this matrix of preventative, monitoring, and therapeutic measures is a powerful rational basis to now support the clinical application of solid organ xenotransplantation.
